# Evaluation of Spin Bias in the Use of Platelet-Rich Plasma for Augmentation of Meniscal Tear Repairs

**DOI:** 10.1177/23259671261471384

**Published:** 2026-08-31

**Authors:** Christina Im, Al-Hassan Dajani, Aidan Marks, Timothy Liu, Christopher Hamad, Thomas J. Kremen, Amanda Honsvall Hoefler, Edward C. Cheung

**Affiliations:** * Department of Orthopaedic Surgery, David Geffen School of Medicine at UCLA, Los Angeles, California, USA; † Yale University, New Haven, Connecticut, USA; Investigation performed at Department of Orthopaedic Surgery, David Geffen School of Medicine at UCLA, Los Angeles, California, USA

**Keywords:** meniscal repair, platelet-rich plasma, biologic augmentation, spin bias

## Abstract

**Background:**

Platelet-rich plasma (PRP) is used as a biologic adjunct for meniscal repair (MR). Systematic reviews and meta-analyses are frequently used to inform treatment decisions, but their abstracts may contain spin bias, overstating benefits and misrepresenting the certainty of the evidence.

**Purpose:**

To determine the prevalence and patterns of spin in systematic reviews and meta-analyses evaluating PRP augmentation of MR.

**Study Design:**

Systematic review; Level of evidence, 4.

**Methods:**

Relevant studies were reviewed and identified in accordance with PRISMA (Preferred Reporting Items for Systematic Reviews and Meta-Analyses) guidelines on July 14, 2025. Each abstract was assessed for 15 predefined spin bias categories, and their corresponding full text was evaluated for methodological quality using A Measurement Tool to Assess Systematic Reviews, Version 2 (AMSTAR 2). General information of the studies was also extracted. Descriptive statistics were used to summarize the incidence of spin bias types and AMSTAR 2 ratings and presented as percentages of total possible responses. Study characteristics, spin bias, and AMSTAR 2 associations were assessed using nonparametric statistical tests, with statistical significance set at *P* < .05.

**Results:**

Screening of 1047 records yielded 16 relevant studies that were included in this analysis. At least 1 form of spin bias was identified in 15 out of 16 (93.8%) of abstracts. The most frequent spin bias types included type 14 (failure to report a wide confidence interval of estimates), type 11 (conclusion focuses selectively on statistically significant efficacy outcome), and type 3 (selective reporting of or overemphasis on efficacy outcomes or analysis favoring the beneficial effect of the experimental intervention), which each appeared in 43.8% of studies. AMSTAR 2 ratings were critically low in 68.8% of studies. No significant associations emerged between study characteristics, spin type, or AMSTAR 2 (all *P* > .05).

**Conclusion:**

The study demonstrated that abstract-level spin bias is highly prevalent in reviews of PRP augmentation for MR, often alongside low methodological confidence. More transparent abstract reporting is needed to ensure clinicians accurately interpret the strength and uncertainty of the available evidence. Reducing spin may help clinicians avoid overestimating PRP benefits when counseling patients and selecting treatments.

Meniscal tears are among the most common orthopaedic injuries with incidence rates of approximately 60 per 100,000 people in the United States.^16,31,47^ Meniscal tears vary significantly in terms of tear morphology, length, location, and proximity to the vascularized “red zone,” and can overall be categorized as either traumatic or degenerative.^4,11^ Typically, without significant displacement, non-root meniscus tears are typically treated with initial nonoperative management.^4^ In patients without advanced joint degeneration who do not improve after nonoperative treatment, a diagnostic arthroscopy is performed to determine if the meniscus tear pattern is amenable to meniscus repair (MR) versus debridement.^12,20,49^ Accumulating clinical evidence shows that loss of meniscal tissue increases tibiofemoral contact stresses and may accelerate degenerative arthritis, making joint-preserving MR the preferred option when feasible.^3,6,22,24,33,34,50^ Despite efforts to preserve meniscal tissue, MR still has a substantial risk of failure with some studies reporting rates up to 48%.^43^ This high rate has been attributed to reduce healing capacity caused by limited vascularity.^2^

The failure rate of meniscal repair (MR) has prompted clinicians to explore biologics as augmentation agents to improve the chances for repair success.^17,50^ Platelet-rich plasma (PRP), an autologous blood derivative with high concentrations of platelets and growth factors, has emerged as a biologic adjunct with particular clinical interest.^28,37,51^ PRP is commonly administered via intra-articular injection or ultrasound-guided injection directly into meniscal tissue, and it is widely applied for a spectrum of tendon, muscle, ligament, and cartilage disorders.^10,35^ Early clinical studies investigating PRP for augmentation of MR reported significantly reduced failure rates and mild improvements in postoperative patient-reported functional outcomes.^14,36^ However, recent practice guidelines by the American Academy of Orthopaedic Surgeons found that a large portion of these studies were conducted in patients with discoid menisci and that overall evidence was limited due to a high proportion of low-level studies.^1^ Many primary studies are limited by heterogeneity in PRP preparation methods and platelet content, insufficient sample size, and limitations in comparison and control groups.^30,41^ Systematic reviews have also reported mixed conclusions regarding PRP's efficacy, with some reviews reporting more protective effects with augmentation than others.^18,21,22,39^

Systematic reviews and meta-analyses are typically ranked high in the evidence hierarchy because of their aggregation of outcomes from multiple studies, but despite their rigor, they remain susceptible to a form of reporting bias called spin. Spin is often introduced by including small or underpowered primary studies in analysis. Moreover, reviews on PRP for augmentation of MR may be particularly prone to spin, as PRP formulations and delivery often follow institutional custom instead of consistent, evidence-based practice.^9,13^ Given this methodological heterogeneity and the risk of summative research papers to influence practice patterns, assessing potential bias is critical. Accordingly, the prevalence and characteristics of bias should be evaluated systematically to allow clinicians and researchers to identify spin before reaching firm conclusions about PRP augmentation and to guide stronger research on the topic.

The primary purpose of this study is to quantify the prevalence and types of spin reporting bias in systematic reviews and meta-analyses studying PRP as an augment for MR. A secondary aim was to identify study characteristics that were potentially associated with higher rates of spin bias. We hypothesize that spin will be highly prevalent in the abstracts of systematic reviews and meta-analyses.

## Methods

**Figure 1.**
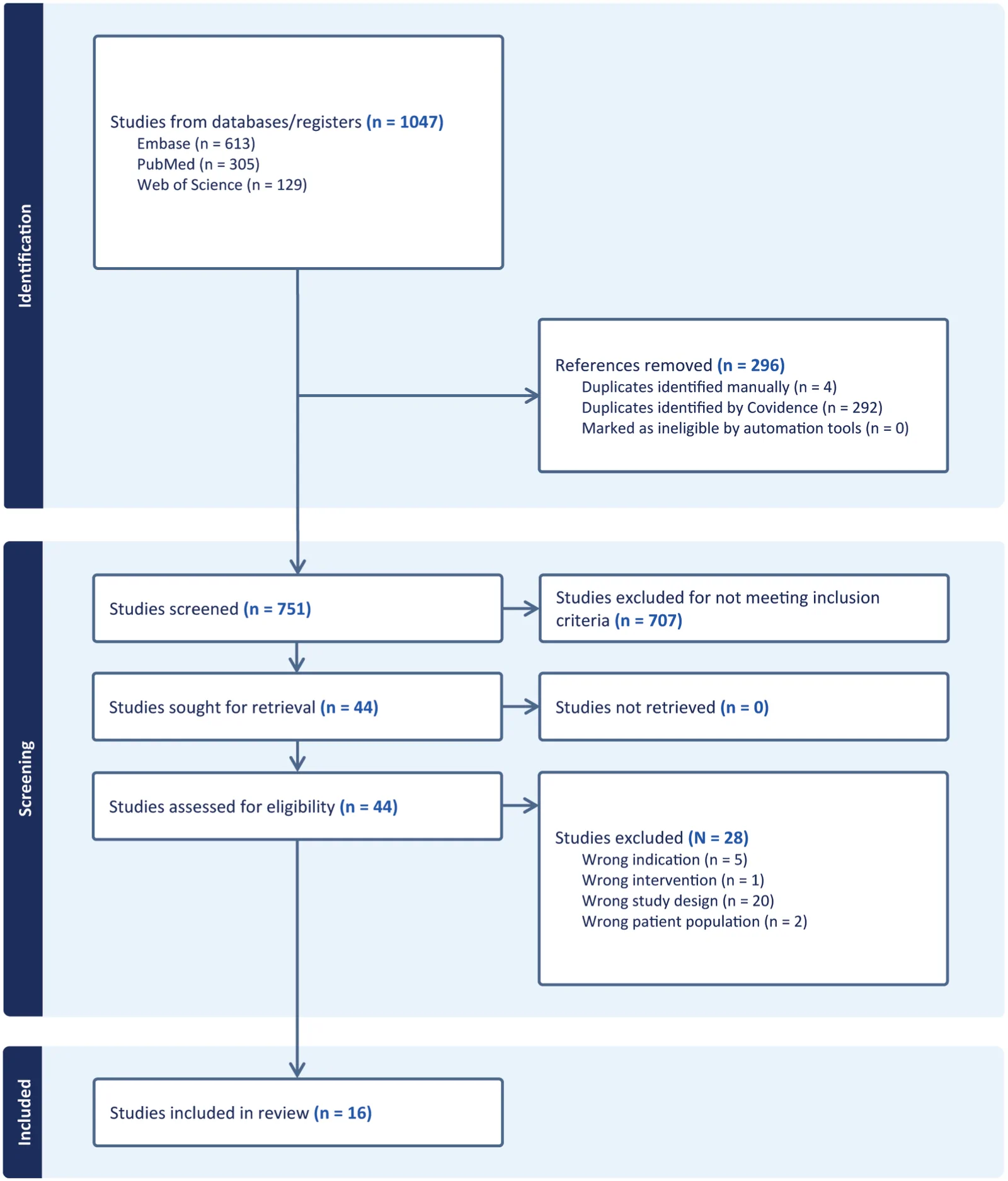
PRISMA (Preferred Reporting Items for Systematic Reviews and Meta-Analyses) flow diagram illustrating screening process and number of studies at each stage of review.

This retrospective review study was completed using PRISMA (Preferred Reporting Items for Systematic Reviews and Meta-Analyses) guidelines. Figure 1 illustrates the screening process.

### Search Strategy

PubMed, Embase, and Web of Science databases were comprehensively searched on July 14, 2025, using the terms (("platelet-rich plasma" OR "platelet rich plasma" OR PRP OR (platelet AND rich AND plasma)) AND menisc* AND ("systematic review" OR "meta-analysis" OR review OR (meta AND analysis))). Initial literature search results were entered into Covidence.

### Eligibility Criteria

Eligible studies included systematic reviews or meta-analyses evaluating PRP injections as an augment to MR. Comprehensive searches were performed from database inception through July 14, 2025, the date the search was conducted. Exclusion criteria included studies that were not peer reviewed, were published in a language other than English, were not in the form of a systematic review or meta-analysis, did not report clinical outcomes, had been retracted or withdrawn, involved nonhuman or cadaveric models, were published without an abstract, or did not have full-text availability.

### Training

Two authors (A.-H.D. and A.M.) underwent training to evaluate the eligible articles for 15 common spin patterns as previously described by Yavchitz et al.^54^ The methodological quality and rigor of each study were evaluated using A Measurement Tool to Assess Systematic Reviews, Version 2 (AMSTAR 2), which has been previously shown to have strong interrater reliability and construct validity.^40^ AMSTAR 2 includes 16 criteria that evaluate whether a review study addressed sources of bias, disclosed funding and conflicts of interest, followed a predefined protocol, and described studies in sufficient detail.

### Data Extraction

Two authors (A.-H.D. and A.M.) independently extracted data from each study, with any discrepancies resolved by a third reviewer (C.I.) using a majority vote. The following data were recorded: study title, authors, year of publication, journal name, level of evidence, study design, funding source, adherence to PRISMA guidelines, journal impact factor, PROSPERO preregistration status, primary outcomes, and secondary outcomes if available. The studies were evaluated for 15 of the most common categories of spin in abstracts as described by Yavchitz et al.^54^ If a spin metric was unable to be characterized using the abstract alone, the authors then referenced the corresponding full text for clarification. Each review study was also evaluated using the previously described and validated 16-item AMSTAR 2 tool.^40^

### Statistical Analysis

Descriptive statistics were used to identify the incidence of 15 spin types. AMSTAR 2 items were coded as yes, partial yes, and no, and are presented as percent of total possible responses. Multivariable models were not used given the small sample size. We evaluated bivariate associations between study characteristics, spin, and AMSTAR 2 using Fisher exact test. Wilcoxon rank-sum tests were used for continuous variables such as Clarivate Impact Factor, Scopus CiteScore, and level of evidence. We also explored Spearman rank correlations against the number of spin categories per abstract. A *P* value of <.05 (ie, α = .05; β = 0.95) was used to determine statistical significance. Statistical analysis was conducted in JMP Pro (Version 18; SAS Institute Inc).

## Results

We initially identified 1047 studies that met search criteria, and after full screening were left with 16 articles that fulfilled all inclusion criteria.^§^ All 16 studies adhered to PRISMA guidelines, while only 5 studies (31.3%) reported external funding^29,42,45,52,55^ and 7 (43.8%) were registered with PROSPERO (University of York).^19,25,29,39,42,45,46^ Fourteen studies (87.5%) had a mean 2024 Clarivate Impact Factor of 2.76 with a range of 1.1-5.4.^‖^ Fifteen studies (93.8%) were indexed to Scopus.^¶^ The CiteScore mean was 4.77 with a range of 2.9 to 9.6.

### Spin Frequency and Statistical Analysis

Every study except 1 had ≥1 form of spin reporting bias present in their abstracts (93.8%)^32^ and 14 out of the 15 spin categories were identified at least once (93.3%). Type 15 (conclusion extrapolates the review's findings to a different population or setting) was the only category to not be reported. Type 14 (failure to report a wide confidence interval of estimates), type 11 (conclusion focuses selectively on statistically significant efficacy outcome), and type 3 (selective reporting of or overemphasis on efficacy outcomes or analysis favoring the beneficial effect of the experimental intervention) were the most common spin categories (7/16; 43.8%). Type 9 (conclusion claims the beneficial effect of the experimental treatment despite reporting bias) and type 5 (the conclusion claims the beneficial effect of the experimental treatment despite a high risk of bias [RoB] in primary studies) were also observed frequently, with type 9 appearing in 6 of 16 (37.5%) and type 5 in 5 of 16 (31.3%). Frequency of each type of spin is reported in Table 1.

**Table 1:** Overall Spin Frequency by Category Within Abstracts of Eligible Manuscripts (N = 16)

Category	Type	Description	Abstracts With Spin, n	Abstracts Without Spin, n
Misleading interpretation	1	The conclusion formulates recommendations for clinical practice not supported by the findings	3^7,39,42^	13^5,9,19,25,29,32,44-46,48,52,53,55^
	2	The title claims or suggests a beneficial effect of the experimental intervention not supported by the findings	2^39,42^	14^5,7,9,19,25,29,32,44-46,48,52,53,55^
	4	The conclusion claims safety based on nonstatistically significant results with a wide confidence interval	2^25,45^	14^5,7,9,19,29,32,39,42,44,46,48,52,53,55^
	9	Conclusion claims the beneficial effect of the experimental treatment despite reporting bias	6^7,29,42,45,53,55^	10^5,9,19,25,32,39,44,46,48,52^
	12	Conclusion claims equivalence or comparable effectiveness for nonstatistically significant results with a wide confidence interval	2^25,53^	14^5,7,9,19,29,32,39,42,44-46,48,52,55^
Misleading reporting	3	Selective reporting of or overemphasis on efficacy outcomes or analysis favoring the beneficial effect of the experimental intervention	7^7,39,45,46,48,53,55^	9^5,9,19,25,29,32,42,44,52^
	5	The conclusion claims the beneficial effect of the experimental treatment despite a high risk of bias in primary studies	5^7,42,45,53,55^	11^5,9,19,25,29,32,39,44,46,48,52^
	6	Selective reporting of or overemphasis on harm outcomes or analysis favoring the safety of the experimental intervention	3^29,45,55^	13^5,7,9,19,25,32,39,42,44,46,48,52,53^
	10	Authors hide or do not present any conflict of interest	1^44^	15^5,7,9,19,25,29,32,39,42,45,46,48,52,53,55^
	11	Conclusion focuses selectively on statistically significant efficacy outcome	7^7,39,45,46,48,52,55^	9^5,9,19,25,29,32,42,44,53^
	13	Failure to specify the direction of the effect when it favors the control intervention	1^7^	15^5,9,19,25,29,32,39,42,44-46,48,52,53,55^
	14	Failure to report a wide confidence interval of estimates	7^7,9,19,25,29,39,45^	9^5,32,42,44,46,48,52,53,55^
Inappropriate extrapolation	7	The conclusion extrapolates the review findings to a different intervention (eg, claiming efficacy of 1 specific intervention although the review covered a class of several interventions)	2^29,55^	14^5,7,9,19,25,32,39,42,44-46,48,52,53^
	8	Conclusion extrapolates the review's findings from a surrogate marker or a specific outcome to the global improvement of the disease	1^5^	15^7,9,19,25,29,32,39,42,44-46,48,52,53,55^
	15	Conclusion extrapolates the review's findings to a different population or setting	0	16^5,7,9,19,25,29,32,39,42,44-46,48,52,53,55^

AMSTAR 2 ratings of review methodological quality are shown in Table 2. All reviews clearly specified their PICO (purpose, intervention, comparison, and outcomes) (item 1: 16/16; 100%) and used a satisfactory method for RoB assessment (item 9: 16/16; 100%). Most studies accounted for RoB when interpreting results (item 13: 14/16; 87.5%) and discussed heterogeneity (item 14: 15/16; 93.8%). Included studies were generally described in adequate detail (item 8: 10/16, 62.5%; 6/16 partial, 37.5%). Frequent shortcomings included studies failing to establish the following: clear protocols with justified deviations (item 2: 6/16 yes [37.5%]; 3/16 partial [18.8%]; 7/16 no [43.8%]); explanation of study design selection (item 3: 5/16 yes [31.2%]; 11/16 no [68.8%]); comprehensive search (item 4: 4/16 yes [25.0%]; 12/16 partial [75.0%]; 0/16 no [0.0%]); list and justification of excluded studies (item 7: 3/16 yes [18.8%]; 0/16 partial [0.0%]; 13/16 no [81.2%]); and reporting funding of included primary studies (item 10: 2/16 yes [12.5]; 14/16 no [87.5%]). Study selection and data extraction using 2 separate reviewers was common but not universal (study selection 13/16, 81.2%; data extraction: 11/16, 68.8%). Most reviews reported potential conflicts of interest (item 16: 14/16, 87.5%). Among the 8 reviews that performed a meta-analysis, appropriate synthesis methods were used in all (item 11: 8/8, 100%), while the impact of RoB on pooled results was assessed infrequently (item 12: 3/8, 37.5%) and publication bias investigations were rare (item 15: 2/8, 25.0%). Overall AMSTAR 2 global ratings were critically low in 11 of 16 (68.8%), low in 3 of 16 (18.8%), and moderate in 2 of 16 (12.5%). No AMSTAR 2 ratings were classified as high.

**Table 2:** AMSTAR 2 Rating of Review Methodological Quality*^*a*^*

AMSTAR 2 Criteria	Yes	Partial Yes	No
1. Did the research questions and inclusion criteria for the review include the elements of PICO?	16^5,7,9,19,25,29,32,39,42,44-46,48,52,53,55^	N/A	0
2. Did the report of the review contain an explicit statement that the review methods were established before the conduct of the review, and did the report justify any significant deviations from the protocol?	6^19,25,39,42,45,46^	3^5,7,29^	7^9,32,44,48,52,53,55^
3. Did the review authors explain their selection of the study designs for inclusion in the review?	5^25,29,39,48,52^	N/A	11^5,7,9,19,32,42,44-46,53,55^
4. Did the review authors use a comprehensive literature search strategy?	4^19,25,45,48^	12^5,7,9,29,32,39,42,44,46,52,53,55^	0
5. Did the review authors perform study selection in duplicate?	13^5,7,9,19,25,32,39,44-46,52,53,55^	N/A	3^29,42,48^
6. Did the review authors perform data extraction in duplicate?	11^5,25,29,32,39,44,46,48,52,53,55^	N/A	5^7,9,19,42,45^
7. Did the review authors provide a list of excluded studies and justify the exclusions?	3^19,45,46^	0	13^5,7,9,25,29,32,39,42,44,48,52,53,55^
8. Did the review authors describe the included studies in adequate detail?	10^5,7,9,19,25,29,45,46,52,53^	6^32,39,42,44,48,55^	0
9. Did the review authors use a satisfactory technique for assessing the RoB in individual studies that were included in the review?	16^5,7,9,19,25,29,32,39,42,44-46,48,52,53,55^	0	0
10. Did the review authors report on the sources of funding for the studies included in the review?	2^5,55^	N/A	14^7,9,19,25,29,32,39,42,44-46,48,52,53^
11. If meta-analysis was performed, did the review authors use appropriate methods for statistical combination of results?*^*b*^*	8^29,32,44,45,48,52,53,55^	N/A	0
12. If meta-analysis was performed, did the review authors assess the potential impact of RoB in individual studies on the results of the meta-analysis or other evidence synthesis?*^*b*^*	3^29,44,45^	N/A	5^32,48,52,53,55^
13. Did the review authors account for RoB in primary studies when interpreting or discussing the results of the review?	14^5,7,9,19,25,29,32,39,42,44-46,52,53^	N/A	2^48,55^
14. Did the review authors provide a satisfactory explanation for, and discussion of, any heterogeneity observed in the results of the review?	15^5,7,9,19,25,29,32,39,42,44-46,52,53,55^	N/A	1^48^
15. If they performed quantitative synthesis, did the review authors carry out an adequate investigation of publication bias (small study bias) and discuss its likely impact on the results of the review?*^*b*^*	2^32,44^	0	6^9,45,48,52,53,55^
16. Did the review authors report any potential sources of conflict of interest, including any funding they received for conducting the review?	14^5,7,9,19,25,29,32,39,42,45,46,48,52,53^	0	2^44,55^

a Total N = 16. N/A, not available; PICO, purpose, intervention, comparison, and outcomes; RoB, risk of bias.

b Studies that performed a meta-analysis (n = 8).

There was no statistically significant correlation between the number of spin categories per abstract and Scopus CiteScore (Spearman *r* = 0.07; *P* = .82) or Clarivate Impact Factor (*r* = −0.11; *P* = .70). Similarly, level of evidence showed no meaningful association (*r* = 0.05; *P* = .86). In bivariate analyses using Fisher exact tests, the presence of any spin was not associated with PRISMA adherence (*P*≥ .99), PROSPERO registration (*P*≥ .99), declaration of external funding (*P*≥ .99), meta-analysis study type (*P*≥ .99), or AMSTAR 2 overall confidence (critically low vs not critically low; *P*≥ .99). Wilcoxon rank-sum tests likewise showed no significant differences between abstracts with versus without spin for CiteScore (median, 4.5 vs 5.3; *P* = .48), Impact Factor (2.5 vs 3.7; *P* = .38), or level of evidence (3.0 vs 2.0; *P* = .82).

## Discussion

The major findings of our study demonstrated that spin bias was present in 93.8% of abstracts of systematic reviews and meta-analyses studying PRP for augmentation of MR. Across all 16 included studies, 14 of the 15 spin bias categories described by Yavchitz et al^54^ were identified. The most frequently observed spin bias subtypes were type 14 (failure to report a wide confidence interval of estimates), type 11 (conclusion focuses selectively on statistically significant efficacy outcome), and type 3 (selective reporting of or overemphasis on efficacy outcomes or analysis favoring the beneficial effect of the experimental intervention), each appearing in 7 out of 16 studies (43.8%). We found no statistically significant relationship between study characteristics, spin type, and AMSTAR 2 confidence levels (all *P* > .05).

We identified a very high prevalence of spin and reporting bias within the abstracts of systematic reviews and meta-analyses on the augmentation of MR using PRP. Given the substantial rate of failure after MR and the growing popularity of biologic augmentation, practicing surgeons may frequently reference the abstracts of systematic reviews to guide their clinical decision-making in an effort to improve patient outcomes. However, it is challenging to draw meaningful conclusions and change practice patterns when those abstracts overstate the clinical value of PRP, even if further qualifying details are provided in the full text. It is essential that research abstracts present an accurate and unbiased summary of the study's objectives, methodology, and findings. Furthermore, we observed that the prevalence of spin did not correlate with traditional markers of review quality, highlighting the importance of critically reviewing abstract claims even when the underlying methodology appears robust. Last, AMSTAR 2 criteria suggest that many existing reviews do not clearly justify their inclusion/exclusion criteria or report the funding of included primary studies, raising further concern about the conclusions drawn.

Multiple investigations have previously documented a high rate of spin in orthopaedic systematic reviews.^8,15,19,23,26^ For example, a systematic review by Kim et al^26^ found that all 17 systematic reviews studying superior capsular reconstruction had ≥1 type of spin. A different study analyzing spin in biodegradable balloon space found that 93.1% of the studies included spin. Similarly, Carr et al^8^ found that 65% of the studies on Achilles tendon ruptures contained spin. However, not all topics show similar rates of spin.

Reddy et al^38^ examined 176 systematic reviews and meta-analyses pertaining to the use of PRP for orthopaedic conditions and identified spin in 28.4% of abstracts, with type 3 and type 5 being the most prevalent. In contrast, we identified spin in 93.8% of abstracts included in our cohort, which is particularly high, even within orthopaedic topics. This suggests that reporting bias is particularly prevalent in reviews analyzing PRP for meniscal tears, which may be due to the comparably few primary studies, high proportion of low-level investigations, and high heterogeneity of relevant studies on this topic with several methodological differences. Reddy et al assessed abstracts only and limited coding to the 9 most common Yavchitz categories through June 2021, whereas our approach examined a narrower clinical domain and used the broader 15-spin categories by Yavchitz et al.^54^ Both of these factors are likely to increase detection, and thus, result in more identified spin.^38^

One of the most frequently cited spin categories in our cohort was type 14 (failure to report a wide confidence interval of estimates), which was reported in 7 studies.^7,9,19,25,29,39,45^ Across published reviews, type 14 is a frequently reported form of spin in abstracts.^26^ In our cohort of PRP-focused reviews, abstracts often conveyed definitive takeaways without presenting confidence intervals, limiting readers’ ability to gauge precision. For example, Haunschild et al^19^ concluded there was “insufficient evidence” that PRP augments MR yet reported only significance statements in the abstract without confidence intervals. Keller et al^25^ listed ranges of revision rates and mixed significance across augmentation strategies but again omitted confidence intervals in the abstract, making imprecision hard to assess. Notably, a minority did include confidence intervals in the abstract; for example, Li et al^29^ reported odds ratio and mean difference with 95% confidence intervals. Given that many included primary studies are small and heterogeneous, failing to display confidence intervals in the abstracts of systematic reviews could lead readers to misinterpret the certainty or significance of the findings. It is prudent to report confidence intervals clearly, as such spin may bias clinicians in favor of or against certain interventions.

Type 11 spin (conclusion focuses selectively on statistically significant efficacy outcome) is also common, and several meta-analyses framed overall treatment efficacy based on statistically significant outcome measures while downplaying neutral findings.^7,39,45,46,48,52,55^ For example, Wang et al^48^ concluded that PRP injection can effectively enhance the efficacy of arthroscopic MR and emphasized lower failure rates, pain scores, and improved active flexion, even though pooled analyses showed no significant differences in their included patient-reported outcomes (International Knee Documentation Committee, Lysholm) between PRP and control groups. Similarly, Sakti et al^39^ summarized their systematic review by stating that MR augmented with PRP has a lower failure rate, without noting in the conclusion that meaningful improvements in patient-reported function were observed in only 1 out of 4 included studies. Selective emphasis on favorable outcomes may lead readers to overestimate the global benefits of PRP augmentation, particularly if actual functional outcomes are inconsistent or not statistically significant.

Type 3 spin (selective reporting of or overemphasis on efficacy outcomes or analysis favoring the beneficial effect of the experimental intervention) was also present in 7 abstracts.^7,39,45,46,48,53,55^ An example of type 3 spin is the study by Trams et al,^45^ which emphasizes improvement in the primary endpoint (pain) with PRP while giving limited attention to other findings. A review of the full text indicated that there were numerous secondary outcomes listed in the review that were not statistically significant, a pattern consistent with selective emphasis. Accordingly, type 3 spin, characterized by omitting key details in the abstract, can mislead readers and skew clinical decision-making.

We identified type 9 spin (conclusion claims the beneficial effect of the experimental treatment despite reporting bias) in 6 abstracts.^7,29,42,45,53,55^ For example, the abstract by Xie et al^53^ framed PRP as beneficial, yet the review did not assess publication bias and lacked protocol registration, limiting confidence that the pooled effect was not inflated by selective reporting. The broader evidence base consistently identified type 9 as a common spin pattern in review abstracts. In a study by Kumaran et al^27^ investigating anterolateral ligament augmentation, type 9 was reported as the most common spin, appearing in 21 of 22 abstracts. Therefore, it is crucial for authors to integrate publication bias assessments and acknowledge their implications directly in the abstract to avoid overstating benefit.

Finally, type 5 spin (the conclusion claims the beneficial effect of the experimental treatment despite a high RoB in primary studies) appeared in 5 of the abstracts and is particularly likely to overrepresent the certainty of otherwise unclear findings about PRP's efficacy in meniscal tear.^7,42,45,53,55^ For instance, Sochacki et al^42^ reported lower failure rates with PRP augmentation in their abstract while simultaneously noting that the included evidence was “mostly of low quality,” raising concern about certainty of PRP's proposed protective effect. In the broader literature on PRP, Reddy et al^38^ also found type 5 to be the single most common spin category across 176 PRP-related reviews, reinforcing how easily affirmative conclusions can outpace the RoB profile of the underlying studies. Thus, authors should be aware of claims in the abstract drawn from lower-quality evidence to avoid biased conclusions.

### Limitations

This review should be interpreted with several limitations. First, although we ultimately included 16 studies, our prespecified criteria narrowed the pool considerably. As a result, the study may have been underpowered to detect associations between abstract-level spin and study characteristics, raising the possibility of type 2 error. In addition, this small sample size makes the high prevalence of spin particularly concerning as there were not enough high-quality data to counter biased conclusions. Second, classifying spin requires human judgment. We used the Yavchitz et al^54^ taxonomy and performed independent, duplicate assessments with consensus resolution to reduce subjectivity, but some variability in interpretation was unavoidable. The AMSTAR 2 appraisals share similar constraints, even with standardized guidance and dual review. Third, our spin evaluation focused on abstracts. While this mirrors how many readers first encounter evidence, reliance on abstracts may not reflect the nuances described in full texts. Finally, the included reviews spanned diverse topics and journals, introducing methodological heterogeneity that could dilute detectable patterns across subgroups.

Spin is highly prevalent in the abstracts of systematic reviews and meta-analyses investigating augmentation of MR using PRP. The 3 most common categories of spin identified were failure to report a wide confidence of estimates, conclusions focusing selectively on statistically significant efficacy outcome, and selective reporting of or overemphasis on efficacy outcomes or analysis favoring the beneficial effect of the experimental intervention. This raises concern about overrepresentation of certainty in heterogeneous and potentially tenuous clinical results. Measures should be implemented to limit abstract-level spin so that abstracts accurately convey the strength of existing clinical evidence on this biologic therapy to guide clinical decision-making and identify gaps for future investigation.

## Conclusion

Our study demonstrated that abstract-level spin bias is highly prevalent in reviews of PRP augmentation for meniscal repair, often alongside low methodological confidence. More transparent abstract reporting is needed to ensure clinicians accurately interpret the strength and uncertainty of the available evidence. Reducing spin may help clinicians avoid overestimating PRP benefit when counseling patients and selecting treatments.

## Footnotes

§ References 5, 7, 9, 19, 25, 29, 32, 39, 42, 44-46, 48, 52, 53, 55.

‖ References 5, 7, 19, 25, 29, 32, 42, 44-46, 48, 52, 53, 55.

¶ References 5, 7, 19, 25, 29, 32, 39, 42, 44-46, 48, 52, 53, 55.
